# On the precision of 6 DoF IMU-LiDAR based localization in GNSS-denied scenarios

**DOI:** 10.3389/frobt.2023.1064930

**Published:** 2023-01-24

**Authors:** Matteo Frosi, Riccardo Bertoglio, Matteo Matteucci

**Affiliations:** Dipartimento di Elettronica, Informazione e Bioingegneria of Politecnico di Milano, Milan, Italy

**Keywords:** GNSS-denied environment, indoor navigation, outdoor navigation, lidar, IMU, SLAM, benchmarking

## Abstract

Positioning and navigation represent relevant topics in the field of robotics, due to their multiple applications in real-world scenarios, ranging from autonomous driving to harsh environment exploration. Despite localization in outdoor environments is generally achieved using a Global Navigation Satellite System (GNSS) receiver, global navigation satellite system-denied environments are typical of many situations, especially in indoor settings. Autonomous robots are commonly equipped with multiple sensors, including laser rangefinders, IMUs, and odometers, which can be used for mapping and localization, overcoming the need for global navigation satellite system data. In literature, almost no information can be found on the positioning accuracy and precision of 6 Degrees of Freedom Light Detection and Ranging (LiDAR) localization systems, especially for real-world scenarios. In this paper, we present a short review of state-of-the-art light detection and ranging localization methods in global navigation satellite system-denied environments, highlighting their advantages and disadvantages. Then, we evaluate two state-of-the-art Simultaneous Localization and Mapping (SLAM) systems able to also perform localization, one of which implemented by us. We benchmark these two algorithms on manually collected dataset, with the goal of providing an insight into their attainable precision in real-world scenarios. In particular, we present two experimental campaigns, one indoor and one outdoor, to measure the precision of these algorithms. After creating a map for each of the two environments, using the simultaneous localization and mapping part of the systems, we compute a custom localization error for multiple, different trajectories. Results show that the two algorithms are comparable in terms of precision, having a similar mean translation and rotation errors of about 0.01 m and 0.6°, respectively. Nevertheless, the system implemented by us has the advantage of being modular, customizable and able to achieve real-time performance.

## 1 Introduction

Positioning represents one of the key problems in robotics. Real-world applications, ranging from small indoor spaces to large outdoor scenes, include unmanned vehicle trajectory planning, 3D reconstruction, or search and rescue during emergencies, and they all require an accurate 6 Degrees of Freedom (6 DoF) positioning of the robot, which can be classified as follows. When the robot moves through an unknown environment, it is referred to as Simultaneous Localization and Mapping (SLAM); here, the robot must build a map of the surrounding area while simultaneously localizing itself inside it. When the robot goes through a known environment, for example, having available a pre-computed map, only the term localization is used, as the robot must only determine its position in the existing map. It is important to understand that in SLAM both map and trajectory of the robot are unknown, and must be computed from scratch; when using the term localization, however, one means that only the trajectory of the robot must be estimated, while the map is already given through some means (e.g., a previous run of a SLAM algorithm).

Although outdoor localization is easily achieved using a Global Navigation Satellite System (GNSS) receiver, the range of situations where the sensor can be used is limited, as environments may contain physical obstacles that shade the GNSS signal (e.g., urban canyons). Also, the attained accuracy with the majority of GNSS systems is inadequate for applications where precise localization is crucial. Over the last decade, many algorithms have been proposed to get precise positioning in real-world indoor and GNSS-denied environments. The idea behind the majority of these works is to use multiple sensors to obtain good estimates of the poses of the robot. The combination of IMU and Light Detection and Ranging (LiDAR) sensors is usually adopted to achieve accurate results and to build detailed 3D maps.

Although the number of systems available for 6 DoF LiDAR positioning is considerable, to the best of our knowledge, there is no work on the attainable positioning precision of these systems in real-world scenarios and with real data Röwekämper et al. (2012). For this reason, the main research question that this paper is answering is not related to the development of a novel localization-based algorithm, but it focuses on the detailed evaluation of existing localization systems. The framework of the paper is in relative localization accuracy, i.e., the localization accuracy referred to a local reference system, which is defined w.r.t., a map. This is done in order to stress the coherence of the systems over multiple runs, but also because ground truth is often hard to obtain in real-world scenarios. As explained later in Section 4, the map is assumed to be built using a SLAM algorithm, and then the accuracy of the localization is computed w.r.t., such map.

Our paper presents the following contributions; first, we provide a literature review of existing 6 DoF LiDAR localization and SLAM systems, highlighting features, advantages and negative aspects. After a brief discussion about which algorithms are more suitable to perform localization, we benchmark those systems with two experimental campaigns, one indoor and one outdoor, evaluating the precision of the results obtained on real-world data, manually collected by us. Although there are plenty of dataset in literature, the majority is gathered with high-end sensor suites, is pre-processed, and it is usually representing the same type of environments (e.g., cities). To truly stress the accuracy of SLAM and precision of localization, we chose to use data collected by us through a low-end sensor suite and without pre-processing it. The two collected dataset represent environments which are not commonly seen in dataset available in literature. Moreover, we took multiple trajectories of more or less the same paths, to perform localization, which is not typical of standard dataset. It is important to stress the fact that, as we want to represent real-world situations as close as possible, we chose scenarios where the ground truth was too challenging to estimate (GNSS-denied, complex structures, and other constraints). This is also the reason why the focus of the work is about relative re-localization accuracy, i.e., w.r.t a previously computed map or trajectory (and not referred to the ground truth, as it is usually done with common dataset).

The rest of the paper is outlined as follows. We first discuss related works in Section 2, to give a brief but detailed insight about existing systems for SLAM and/or localization, describing advantages and disadvantages. Then, in Section 3 we show the sensors used in the experiments and describe the localization algorithms used, along with the adopted evaluation metrics. Follows Section 4, which is dedicated to the validation of the selected systems. Lastly, Section 5 concludes the manuscript.

## 2 Related works

Vehicles and robots are commonly equipped with IMU sensors to measure linear acceleration and angular velocity. These data can be used to calculate the location of the vehicle relative to the starting point. However, the accumulated error will make the localization algorithm unreliable after a few meters. GNSS sensors are commonly used to correct the accumulated error of IMU sensors, in outdoor environments. Nevertheless, this is not possible in GNSS-denied environments. Thus, the scientific community explored alternative approaches exploiting also LiDAR information, which allows to achieve accurate results.

A well-known method for 3D localization and mapping is LiDAR Odometry and Mapping (LOAM) Zhang and Singh (2014). This work aims to divide complex tasks that are typically solved simultaneously using SLAM methods. These algorithms work by optimizing a large number of variables at the same time, resulting in low-drift but high-computational complexity algorithms. LOAM is designed to limit the drift error while achieving real-time performance. Thus, one algorithm performs odometry at a high frequency, but low fidelity, to estimate the velocity of the LiDAR. Another algorithm runs at a lower frequency for fine processing to create a map with point clouds. Mapping is conducted with a method similar to Iterative Closest Point (ICP) Besl and McKay (1992), to produce high-precision maps. Then, the information from velocity estimation and mapping are combined to produce accurate motion estimates. Even if the methods showed relatively low error estimations compared to the length of trajectories, the lack of a loop closure causes noticeable drifts over time, making LOAM unusable for long paths.

LeGO-LOAM Shan and Englot (2018) is a LiDAR odometry and mapping method based on LOAM. The system is divided into five modules. The segmentation module produces a 2D representation of the LiDAR clouds. The obtained range images are then segmented *via* a clustering algorithm, and groups with few points are discarded from the image. This is done in order to perform fast and reliable feature extraction, which is the next step. Features are successively used to match scans for finding the transformation between them in a module called LiDAR odometry. The LiDAR mapping module also uses said features, registering them to a global point cloud map. Finally, the transform integration module fuses the pose estimation results from LiDAR odometry and LiDAR mapping and outputs the final pose estimate. LeGO-LOAM aims to improve the efficiency and accuracy relative to the original LOAM framework. However, as with most of similar approaches, LeGO-LOAM is an odometry and mapping method. Thus, there is not an easy way to adapt the implemented code just for the localization task. A new map is created at every run of the algorithm, and it is not possible to localize the robot on an already created map.

PoseMAP is a localization method designed for 3D LiDARs. Based on the matching of extracted distinctive 3D features in point clouds, PoseMAP is thought for lifelong localization. It has been tested for 18 months through a mix of human-made structured and off-road unstructured environments without a single failure Egger et al. (2018). Even the changes that occurred in the environment during this long period did not affect the localization accuracy. Indeed, the system can update the map and extend it in case of newly seen environments. However, the authors did not make the code available to the public.

BLAM! is an open-source software package for LiDAR-based real-time 3D localization and mapping. BLAM!^1^ was developed by Erik Nelson from the Berkeley AI Research Laboratory. However, the author provides neither a scientific paper nor a guide describing how the algorithm works. Thus, the only way to know how this method works is to read the source code entirely and thoroughly test it.

GICP-SLAM Ren et al. (2019) is a 3D LiDAR SLAM method thought for indoor and harsh environments, like mines. As the name suggests, it performs scan matching *via* the GICP algorithm Segal et al. (2009). GICP-SLAM is a graph-based SLAM Grisetti et al. (2010) method; thus, it performs optimization of a graph, known as pose graph, where nodes are the robot poses, and edges are the transformations between the poses. Also, other constraints than transformations can be added to the pose graph. The general framework of GICP-SLAM is the following. The input point cloud from the 3D LiDAR is sent to three modules. One is in charge of plane detection to exploit this as an additional constraint. The second performs LiDAR odometry by matching two successive scans *via* GICP. The last one is in charge of handling loop detection constraints. After the robot motion between scans is calculated, another module refines it by scan matching the current scan with the map. All the constraints and the refined transformations are sent to the Pose Graph Optimise module that outputs the final pose result that is used to build the map. Also in this case, the authors did not make the code available to the public, making GICP-SLAM unusable.

HDL Koide et al. (2019), consists of a 3D LiDAR-based SLAM algorithm for long-term operations. The system comprises two main modules, one dedicated to offline mapping, the other for real-time localization. The mapping is done with a graph-based SLAM algorithm that exploits a scan matching technique, similarly to GICP-SLAM. Being a SLAM algorithm, a loop detection and closure procedure has been implemented. Moreover, in order to compensate for the accumulated rotational error, the authors introduced a ground plane constraint, to build consistent maps in long-term scanning processes. The localization algorithm is implemented as an Unscented Kalman Filter (UKF) Wan and Van Der Merwe (2000), combining the information from the scan matching of the current scan to the previously constructed map and a prediction step, which uses the angular velocity and linear acceleration from an IMU sensor.

ART-SLAM Frosi and Matteucci (2022) ^2^ is also a 3D LiDAR-based SLAM algorithm, similar to HDL, implemented by the corresponding author of this paper. The mapping follows the same Graph SLAM approach, allowing for different context-based scan-matching techniques (e.g., ICP, GICP), while also performing fast and efficient loop detection, making the whole method scalable and suitable for real-time applications. ART-SLAM includes other differences w.r.t., HDL, including parallelized point cloud pre-processing and improved floor detection. Being ART-SLAM easy to extend and to work with, we implemented a localization module, named ART-SLAM LOC, to be part of the evaluation presented in this article. The module works the same way as the HDL localization module, with some minor performance improvements, such as ground removal from both map and input scans, for efficient pose correction, and up-sampled prediction. Unlike HDL, both ART-SLAM and ART-SLAM LOC are not bound to any framework and have a high degree of modularity, making them customizable and easy to improve.

## 3 Materials and methods

In Section 2, we have shown how 6 DoF positioning for indoor and GNSS-denied scenarios can be achieved with precision using LiDAR sensors, integrated with other data sources (e.g., IMU). With the purpose of understanding and evaluating existing algorithms for LiDAR-based 6 DoF localization, we decided to perform two experimental campaigns, collecting data from an indoor space and from a GNSS-denied outdoor environment, to evaluate the precision of these methods. Our intent is also to contribute to the literature, as little information is available regarding this context of navigation and positioning (GNSS-denied, 6 DoF LiDAR-based) in real-world environments. In this section, we first describe the sensors we used in our experimental campaigns. Then, we compare the presented systems to choose which of them is suitable to perform both SLAM and localization, and lastly we detail the evaluation metrics adopted.

### 3.1 Materials

The sensor suite used in our experiments to perform all the mapping and localization tasks consisted of an Ouster OS-1 *sensor* that provided both LiDAR and IMU data. The LiDAR component has a range resolution of 1.2 cm, a vertical resolution of 64 beams and a horizontal resolution of 1024. The vertical FOV is 33.2° and the horizontal FOV is 360°. The angular sampling accuracy is ±0.01°, both vertical and horizontal, and the rotation rate is configurable at 10 Hz or 20 Hz. We configured the sensor to retrieve data at 20 Hz, thus having point clouds of about 65 K points. The IMU component gathers higher frequency data (100 Hz), allowing for a fast localization. We mounted the sensor on a four-wheeled cart, which was manually pulled through selected locations of the experiments area, trying to keep the same speed.

### 3.2 Methods

To find the most suitable approaches to test for benchmarking, we analyzed the characteristics of the systems discussed in Section 2. In Table 1, we report a summary of the 3D LiDAR odometry and mapping methods reviewed in the previous section. We indicate the framework adopted in each work, the type and purpose of the system, whether a localization algorithm is provided, and whether the code is available. SLAM methods differ from Odometry and Mapping systems because the firsts add loop closure.

**TABLE 1:** Summary of the 3D localization and mapping approaches. Methods are also compared based on whether they adopt the Robot Operating System (ROS) Quigley et al. (2009) as framework or not.

System	Framework	Purpose	Localization	Code
LOAM Zhang and Singh (2014)	ROS	Odometry∖mapping	No	Yes
LeGO-LOAM Shan and Englot (2018)	ROS	SLAM	No	Yes
PoseMAP Egger et al. (2018)	None	Localization	Yes	No
BLAM!	ROS	SLAM	No	Yes
GICP-SLAM Ren et al. (2019)	None	SLAM	No	Yes
HDL Koide et al. (2019)	ROS	SLAM	Yes	Yes
ART-SLAM Frosi and Matteucci (2022)	None	SLAM	Yes	Yes

For the testing phase, we opted for the approach of Koide et al. (2019) and ART-SLAM Frosi and Matteucci (2022) (including the localization module implemented by us). Both are SLAM algorithms, and, as already said, they can rely on a drift correction procedure using loop detection and closure. This can guarantee long-term mapping operations for large-scale outdoor environments and very accurate results in indoor and short scenes. Both are graph-based, meaning that trajectories and maps can be efficiently computed, manipulated, and stored. Both have the best performance relative to other state-of-the-art 6 DoF LiDAR SLAM approaches. They also both have mapping and localization modules, allowing us to benchmark the positioning using the maps generated by the algorithms themselves.

The SLAM components of both algorithms present great accuracy, even on long trajectories, w.r.t., other methods. A detailed comparison between different SLAM algorithms [including LOAM Zhang and Singh (2014), LeGO-LOAM Shan and Englot (2018), HDL Koide et al. (2019) and ART-SLAM Frosi and Matteucci (2022)] can be found in Frosi and Matteucci (2022), in which it is shown that both HDL and ART-SLAM prove to be accurate in determining the trajectory of a robot, and consequently, in building a high-fidelity map of the environment, to be used later, for localization. When considering datasets collected at higher frequency, i.e., the ones used in this paper, the accuracy and precision of the systems further increase, hence making them suitable methods for benchmarking.

Lastly, the localization algorithm is implemented as an Unscented Kalman Filter Wan and Van Der Merwe (2000), in both methods. While IMU data is used in the prediction step, the correction phase is performed by aligning a LiDAR scan with a global map, performing scan-to-map registration. This way, errors in the correction step are independent of each other and not related temporally: each scan-to-map alignment is affected by its own error. The overall error thus depends on the distribution of the error in the scan matching between the current scan and the map, meaning on the global accuracy of the map itself. Both the current scan and the map do not depend on the length of the trajectory. To be more precise, the prediction step could vary with the length of the trajectory, based on the distance of the initial guess for the minimization of the scan-to-map alignment; nevertheless, the correction phase is bounded by the residual error of the previous scan and the error in one step prediction of the IMU. Both errors are one-step errors, and they get reset at each alignment, making both HDL and ART-SLAM LOC viable solutions (also in terms of memory usage) when performing localization on trajectories of any length and duration.

To evaluate the selected algorithms, we estimated the localization accuracy on specific poses by manually re-positioning the system in the same spots, w.r.t., a previous trajectory (usually the first, for each experimental campaign). Thus, we named this error “re-localization” error. In our indoor experiments, we recorded a first trajectory by stopping the system in specific marked poses. Then, we recorded a second trajectory, reasonably close to the first one, stopping more or less at the same specific positions. A ground truth acquisition system, i.e., an OptiTrack^3^ motion capture system, was used in the indoor experiments to compensate for the difficulty of manually placing the sensor exactly in the same positions. The system exploits custom infrared cameras to localize special reflective markers attached to the robot being tracked, which must be in the field of view of at least three cameras of the OptiTrack system. For this reason, the OptiTrack is not easily deployable in large areas, and it can be used only in restricted locations (in our case, a very limited part of the indoor environment available to us). When the reflective markers on the robot are correctly detected, the system retrieves the 6 DoF position of the rigid body formed by the markers.

In the indoor case, the re-localization error of the systems was measured on a set of poses by removing any bias due to incorrect re-positioning, using the OptiTrack. In the outdoor scenario, i.e., an O&G refinery, the ground truth was not available, making it difficult to collect the positioning ground truths, due to the presence of many physical obstacles (e.g., pipes and trusses), which, due to their density and placement, do not allow the usage of GNSS signals (typical of similar environments). Thus, in the outdoor case, we expect the re-localization error to include the error of manually re-positioning the sensor in the same poses.

**FIGURE 1:**
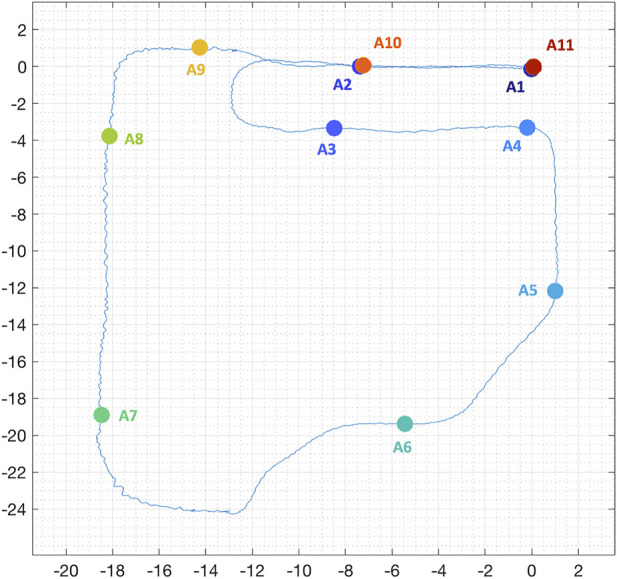
Position, in meters, of the selected eleven locations in the outdoor experiments, used for re-localization. The underlying blue line represents the first estimated trajectory with one of the systems.

As mentioned above, we tried to re-position the robot in the same spots, trying to record trajectories as similar as possible one to the other. In particular, we selected ten locations for the indoor scenario and eleven locations for the outdoor experiment. The spots were selected such that the distance traveled between two of them is more or less always the same. Moreover, the locations were placed in particular areas of interest in both scenarios (e.g., beginning and end of the trajectory or before and after a curve). Figure 1 represents the positions of the eleven locations in the outdoor experiment, used for re-localization. It is important to notice that different areas overlap. Differently from the majority of works, we selected the re-localization positions not just in terms of spatial configuration (equally distant, and nearby zones of interest, such as curves), but also temporal, as each location is uniquely determined in all trajectories. To better explain this, for each trajectory recorded we stopped the cart in proximity of the testing locations, and checked the associated timestamps. All trajectories have then been matched using these timestamps, in order (e.g., first timestamps of all trajectories correspond to testing area A1). This approach allows us to decouple the physical locations from the order of visiting, resulting in a more coherent benchmarking.

**FIGURE 2:**
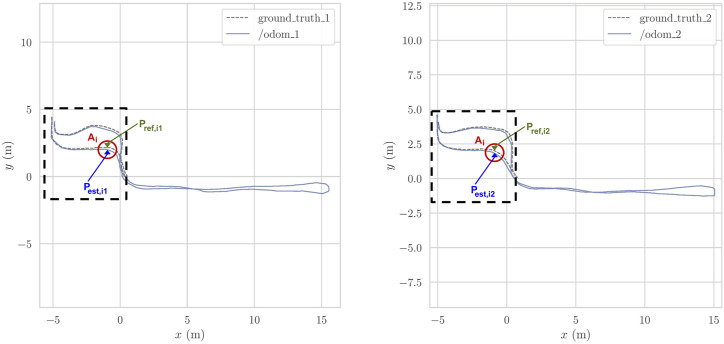
Two indoor trajectories associated to the indoor re-localization error experiments; the OptiTrack area is black-dashed contoured. On the left the first path, while on the right the second trajectory.

To measure the re-localization error, we adopted the metrics used in the most popular benchmarking works like Kümmerle et al. (2009); Geiger et al. (2012); Sturm et al. (2012); Fontana et al. (2014). In the following, we provide the mathematical formulation of the problem. Figure 2 shows the plots of two indoor example trajectories from our indoor experiment, with the following conventions. In creating both trajectories, we recorded the estimated poses given by HDL and ART-SLAM, and the ground truth poses from the OptiTrack. The experiments were carried out manually pulling the cart throughout a laboratory, starting from the OptiTrack area, going outside that tracked area, then coming back to conclude the path (see Figure 2). We decided to go beyond the tracked area to make a longer trajectory, and further stress the SLAM algorithms, while trying to make the paths as similar as possible. The idea was to estimate the accuracy of the localization algorithms when the system is placed in the same location, between different runs of the algorithm. Thus, the re-localization error tells us how the system is consistent in giving the same pose over time, i.e., how the SLAM components of the evaluated systems are coherent over multiple runs.

For each area *A*_*i*_, and for each couple of trajectories, we have manually picked a pose *P*_*est,ij*_ estimated by the localization algorithm; where *i* ∈ {1…*n*_*poses*_} is the area index, and *j* ∈ {1, 2} is the trajectory index. A pose *P* is a vector that contains position information along the three directions *x*, *y*, *z*, and orientation as Euler angles, composed of the three elements *roll*, *pitch*, *yaw*. For each area, we associate the corresponding ground truth pose *P*_*ref,ij*_, if it exists, as the one closer in time according to the timestamps of the messages. Given that the OptiTrack produces data from 50 to 100 times faster than the odometry algorithm, we are sure to find very close correspondences in time. We tried to manually park the system in the same areas in both trajectories; nevertheless, it is nearly impossible to manually re-position a cart in the same place. We estimate this difference in re-positioning using the OptiTrack poses, when available.

Mathematically speaking, we computed the transformation matrix *T*_*ref,i*_ between ground truth poses for each area *A*_*i*_ as

$$T_{\mathrm{ref,i}}=transm\left(P_{\mathrm{ref},i\text{1}},P_{\mathrm{ref},i\text{2}}\right)$$

and the transformation matrix *T*_*est,i*_ between estimated poses for each area *A*_*i*_ as

$$T_{\mathrm{est,i}}=transm\left(P_{\mathrm{est},i\text{1}},P_{\mathrm{est},i\text{2}}\right)$$

where *transm*() is a function that gives as output the transformation matrix necessary to express the second argument of the function in the reference system of the first argument (as both are 3D poses).

Then, we calculated the error matrix *E*_*i*_ as the inverse composition between the two transformations *T*_*est,i*_ and *T*_*ref,i*_

$$E_{i}=inv\left(T_{\mathrm{ref,i}}\right)*T_{\mathrm{est,i}}$$

where *inv*() is the usual inversion matrix operator. Intuitively, the matrix *E*_*i*_ tells us the difference in translation and rotation between the two transformations *T*_*est,i*_ and *T*_*ref,i*_. Finally, we estimated the translation and rotation errors, *trans*_*e*_*i*_ and *rot*_*e*_*i*_, respectively, with the following metrics:

$$trans\text{\_}e_{i}=\|trans\left(E_{i}\right)\|$$

where the function *trans*() extracts the translation part of a given transformation matrix,

$$rot\text{\_}e_{i}=|angle\left(rotm2axang\left(E_{i}\right)\right)|$$

and the function *rotm*2*axang*() converts the rotation matrix extracted from a given transformation matrix, to the corresponding representation of the axis angle, and the function *angle*() extracts the angle from an axis-angle representation. The axis-angle representation is just one of many ways to represent rotations together with quaternions, Euler angles, rotation matrices, and many others. The advantage of the axis-angle representation consists in condensing the rotation in one single number instead of having three components, namely *roll*, *pitch*, *yaw*, as with Euler angles. A much more detailed explanation of 3D rotation error metrics can be found in Huynh (2009), including also the various representations.

In the outdoor scenario, since there was no ground truth, we only calculated the transformation matrix *T*_*est,i*_ between the estimated poses *P*_*est,i1*_ and *P*_*est,i2*_, for each area *A*_*i*_, considering it as error matrix. Obviously, this interpretation does not take into account for possible re-positioning mistakes, which are then included in the whole re-localization error estimation procedure.

## 4 Results

### 4.1 Indoor experiment

In this section we report the information relative to the first, indoor, experiment, corresponding to a large lab room. First, we give some generalities about the setup, data collection and registered trajectories. Then, we show the comparison between the localization obtained through HDL and ART-SLAM, discussing both translation and rotation errors of the second trajectory w.r.t., the map obtained from the first one.

#### 4.1.1 Setup and data collection

**FIGURE 3:**
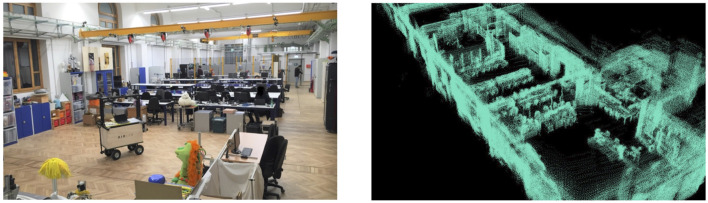
Indoor area of the experimental campaigns. It consists of a long laboratory (left panel) with many obstacles, visible in the map, as a point cloud (right panel), obtained with HDL Koide et al. (2019).

As indoor scenario, we used the main large room of our laboratory (AIRLab, Politecnico di Milano, Italy), represented in Figure 3. The cart, described in Section 3.1 and visible in the left panel, was pulled along two trajectories, which characteristics are listed in Table 2. Overall, ten areas *A*_*i*_, *i* ∈ {1…10} have been selected in the room, to be used in the evaluation of the algorithms.

**TABLE 2:** Information about the two trajectories of the indoor experiment. The first one is used to build a 3D map of the environment using SLAM, while the other allows to evaluate the localization algorithms.

Feature	Trajectory 1	Trajectory 2
Duration	308 (s)	306 (s)
Estimated length	49.71 (m)	48.31 (m)
Estimated mean speed	0.28 (m/s)	0.24 (m/s)
Estimated max speed	0.52 (m/s)	0.50 (m/s)
# Laser scans	6167	6121
# IMU samples	30832	30606

#### 4.1.2 Indoor localization

Using the first trajectory *traj*_1_ with both SLAM methods, ART-SLAM and HDL, we reconstruct an accurate representation of the room, in the form of a dense cloud. As example, the right panel of Figure 3 shows the map obtained with HDL, which contains about 1 M points, while the number of acquired points sums up to 404 M elements. The dimension and accuracy of the obtained map (and the same can be said for ART-SLAM) is, along with the explanation done in Section 3.2, due to the algorithm being graph-based. For this reason, not all the laser scans are saved and used to build the map, but just the most relevant (e.g., after a certain distance has been traveled). This allows to run even long trajectories, while maintaining high accuracy and being memory friendly, features that other algorithms in literature do not have.

**FIGURE 4:**
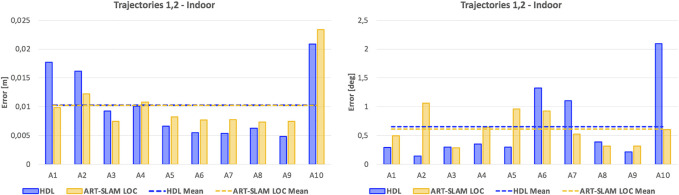
Re-localization error of the indoor experiment, consisting of both translational and rotational components, using HDL Koide et al. (2019) and ART-SLAM Frosi and Matteucci (2022).

**FIGURE 5:**
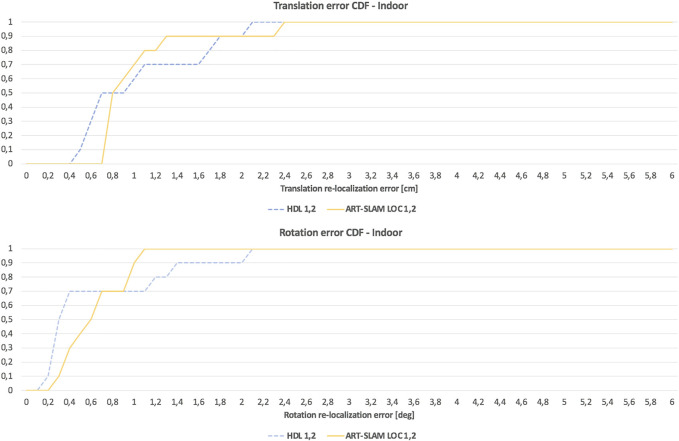
CDF of the re-localization errors of the indoor experiment, consisting of both translational and rotational components, using HDL Koide et al. (2019) and ART-SLAM Frosi and Matteucci (2022).

As stated in Section 3.2, we use the first trajectory to build the 3D map. Then, both the first and the second trajectories are used for re-localization, obtaining multiple estimates of the robot pose (one for each laser scan). The translation re-localization errors for all the testing areas are represented in the left image of Figure 4, while the rotation re-localization errors can be seen in the right panel. Both HDL and ART-SLAM LOC achieve superior accuracy, resulting in errors in the magnitude of centimeters for the translation and 10th of degree angle for the rotation. While the accuracy is similar, ART-SLAM (with its localization module) has the advantage of being real-time [or even faster, see Frosi and Matteucci (2022)], modular, and a zero-copy software. Moreover, Figure 5 shows the cumulative distribution function (CDF) over both translation and rotation re-localization errors. We set the reachable values to 6 cm and 6 degrees, as these correspond to the maximum errors that were seen in both indoor and outdoor experimental campaigns, to allow a fair comparison between all CDFs and without loss of generality.

Despite the low number of samples (the ten areas considered for re-localization), both Figures 4, 5 confirm that the length of a trajectory, or the acquisition time, are not correlated to the attained precision. Both tested systems rely on UKF localization, with a correction step performed *via* scan-to-map point cloud alignment; this way, possible localization errors are bounded by the residual error of the previous scan and the error in one step prediction of the IMU. This was expected, as both errors correspond to one single step in the UKF algorithm and they get reset at each alignment, making both systems scalable, but also independent from time (errors depend only on the characteristics of the considered locations).

Lastly, we give a brief comparison about the processing time of both algorithms when performing localization [as the comparison for the SLAM parts is already done in Frosi and Matteucci (2022)]. ART-SLAM LOC processes inputs as fast as the data acquisition rate of the LiDAR (as the correction step is the bottleneck of the system, being dependent on scan matching between a point cloud and a whole 3D map), making it able to run real-time. HDL, on the other hand, is slower, working at a lower processing rate.

### 4.2 Outdoor experiment

In this section we describe the second experiment, which is outdoor, in an oil refinery. As before, first we give some generalities about the setup, data collection and registered trajectories. After that, we show the comparison between the localization obtained through HDL and ART-SLAM, on all the five trajectories, discussing, once again, both translation and rotation errors w.r.t., the map obtained with the first trajectory.

#### 4.2.1 Setup and data collection

**FIGURE 6:**
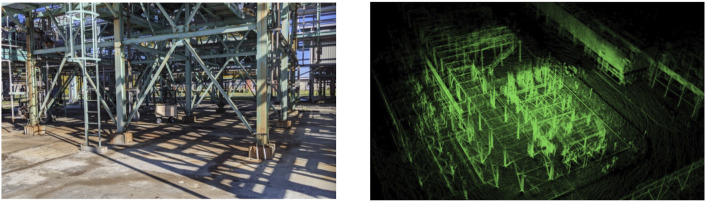
Outdoor area of the experimental campaigns. It is the Eni Centro Olio area at Trecate (NO), Italy. This refinery was traversed to record five trajectories. The left panel shows the environment, while the right image represents the 3D map, obtained with ART-SLAM Frosi and Matteucci (2022).

**FIGURE 7:**
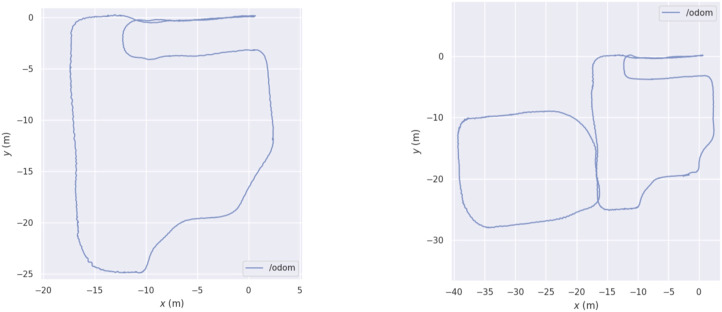
Difference between the first and fourth trajectories of the outdoor experimental campaign.

The chosen outdoor space for testing the mapping and re-localization algorithms is the Eni Centro Olio area at Trecate (NO), in Italy, visible in the left panel of Figure 6. In this experiment, which represents a typical GNSS-denied scenario, we pulled the cart along five trajectories, which characteristics are listed in Table 3. Differently from the indoor scenario, where the two trajectories where almost identical, in the outdoor experiment, the fourth trajectory is much longer than the one used to create the map, as it extends far from the designated area. Figure 7 left and right panels correspond, respectively, to trajectories 1 and 4, making visible the difference between the two paths. Overall, eleven areas *A*_*i*_, *i* ∈ {1…11} have been selected in the outdoor area, to be used in the evaluation of the algorithms.

**TABLE 3:** Information about the five trajectories of the outdoor experiment. The first one is used to build a 3D map of the environment using SLAM, while the other allows to evaluate the localization algorithms.

Feature	Trajectory 1	Trajectory 2	Trajectory 3	Trajectory 4	Trajectory 5
Duration	489 (s)	454 (s)	403 (s)	552 (s)	519 (s)
Estimated length	127.79 (m)	122.58 (m)	123.73 (m)	208.58 (m)	126.69 (m)
Estimated mean speed	0.26 (m/s)	0.27 (m/s)	0.31 (m/s)	0.38 (m/s)	0.24 (m/s)]
# Laser scans	9779	9084	8051	11043	10387
# IMU data samples	48888	45416	40251	55208	51930

#### 4.2.2 Outdoor localization

Using again the first of the five trajectories, *traj*_1_, we are able to achieve a full representation of the environment, as a dense 3D map. Figure 6 shows the map obtained through ART-SLAM, which contains about 1 M points, the same as the one obtained during the indoor experiment. Stressing again the importance of using graph-based algorithms for SLAM, it is remarkable how, comparing it with the indoor scenario, the outdoor experiment maintains the same degree of accuracy, even if the trajectory is much longer and the area containing it is larger. This is also a confirmation that the selected systems are scalable and efficient.

**FIGURE 8:**
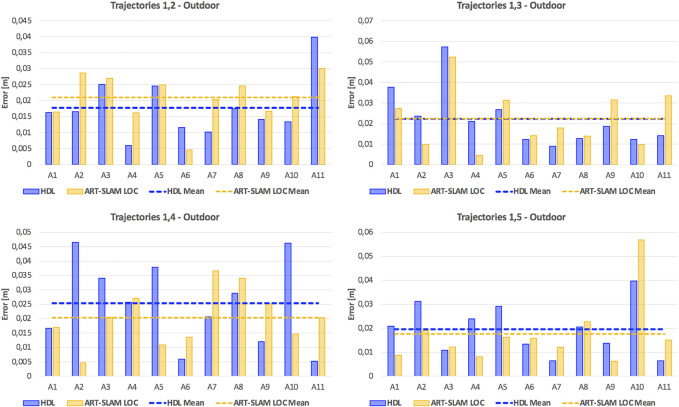
Translation re-localization errors of the outdoor experiment, using HDL Koide et al. (2019) and ART-SLAM Frosi and Matteucci (2022). The accuracy of both systems is noticeable.

**FIGURE 9:**
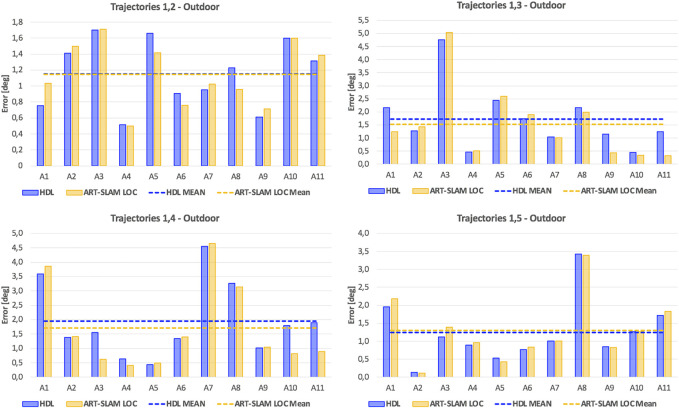
Rotation re-localization errors of the outdoor experiment, using HDL Koide et al. (2019) and ART-SLAM Frosi and Matteucci (2022). Again, both methods prove to achieve superior precision.

Once again, as stated in Section 3.2, we use the first trajectory to build the 3D map. Then, the five trajectories are used for re-localization, obtaining multiple estimates of the robot pose (one for each laser scan). Figures 8, 9 show, respectively, the translation and rotation re-localization errors for all the four pairs of trajectories (1-2, 1-3, 1-4, and 1-5), for all the testing areas (Figure 6). As for the indoor experiment, both HDL and ART-SLAM, with the localization module, are proven to be accurate.

**FIGURE 10:**
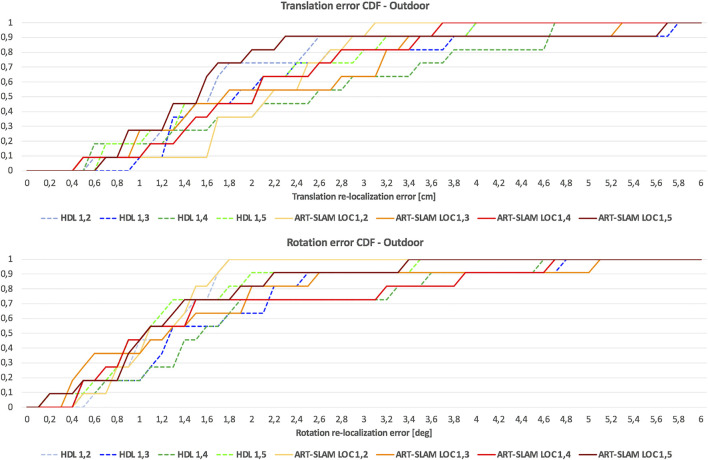
CDF of the re-localization errors of the outdoor experiment, consisting of both translational and rotational components, using HDL Koide et al. (2019) and ART-SLAM Frosi and Matteucci (2022).

As it was done for the indoor experimental campaign, Figure 10 shows the cumulative distribution function (CDF) over both translation and rotation re-localization errors. Stressing what stated before, to allow a fair comparison of CDFs for both indoor and outdoor scenarios, we set a maximum value of 6 cm for the translation component, and 6 degrees for the rotation part. Even in this case, it can be noticed that the values distribute in a uniform-like way over a possible range of errors, also confirming that errors are not time-correlated, but depend only on the physical characteristics of the environment.

Differently from the indoor scenario, errors are slightly larger, probably due to the noisy measurements, typical of outdoor environments and for the unavailability of ground truth compensating re-positioning errors (differently from the indoor experiment, where these errors were corrected using the OptiTrack). Moreover, here the path traversed is longer than the one used in the previous scenario, showing how 3D laser-based positioning is reliable even in larger areas. Lastly, the error distribution confirms that the precision of the systems does not depend on the size or duration of the trajectories, as stated before, and the processing time comparison results in the same considerations done for the indoor experiment (ART-SLAM working in real-time, differently from HDL, which has slightly worse performance).

## 5 Conclusion

In this paper we presented a review of 3D LiDAR systems used for robot positioning, highlighting the methods most suitable for SLAM and localization in GNSS-denied real-world scenarios. Based upon code availability, performance, and purpose, we then selected two systems to benchmark, HDL and ART-SLAM (including the localization module implemented by us), using datasets that we collected thanks to a custom sensor suite (Ouster OS-1 for both IMU and laser scans). The benchmarking was done on two real-world scenarios, both located in Italy: a long room of a laboratory, as indoor case, and an area corresponding to a part of an oil refinery, as GNSS-denied outdoor environment (due to metal pipes and trusses).

We evaluated the algorithms comparing the estimated re-localization positions w.r.t., previously computed maps using the translation and rotation errors as metrics for comparison. We showed that both tested methods achieve high precision, with ART-SLAM being faster both for mapping and localization. These results make them suitable for real-world applications, such as autonomous driving.

## Data Availability

The datasets presented in this article are not readily available because the dataset cannot be given, as bounded by a contract with a company, as mentioned in the manuscript. Requests to access the datasets should be directed to no one, as the dataset cannot be disclosed.

## Footnotes

1 The source code can be found at this link: https://github.com/erik-nelson/blam.

2 The source code of the mapping module can be found at this link: https://github.com/MatteoF94/ARTSLAM.

3
https://optitrack.com/motion-capture-robotics/
